# Willow and Herbaceous Species’ Phytoremediation Potential in Zn-Contaminated Farm Field Soil in Eastern Québec, Canada: A Greenhouse Feasibility Study

**DOI:** 10.3390/plants12010167

**Published:** 2022-12-30

**Authors:** Alexandre Licinio, Joan Laur, Frederic E. Pitre, Michel Labrecque

**Affiliations:** Institut de Recherche en Biologie Végétale, Université de Montréal, Jardin Botanique de Montréal, Montréal, QC H1X 2B2, Canada

**Keywords:** phytomanagement, Zinc, co-cropping, agrobiodiversity

## Abstract

Phytoremediation shows great promise as a plant-based alternative to conventional clean-up methods that are prohibitively expensive. As part of an integrated strategy, the selection of well-adapted plant species as well as planting and management techniques could determine the success of a long-term program. Herein, we conducted an experiment under semi-controlled conditions to screen different plants species with respect to their ability to phytoremediate Zn-contaminated soil excavated from a contaminated site following a train derailment and spillage. The effect of nitrilotriacetic acid (NTA) application on the plants and soil was also comprehensively evaluated, albeit we did not find its use relevant for field application. In less than 100 days, substantial Zn removal occurred in the soil zone proximal to the roots of all the tested plant species. Three perennial herbaceous species were tested, namely, *Festuca arundinacea*, *Medicago sativa,* and a commercial mix purposely designed for revegetation; they all showed strong capacity for phytostabilization at the root level but not for phytoextraction. The Zn content in the aboveground biomass of willows was much higher. Furthermore, the degree of growth, physiological measurements, and the Zn extraction yield indicated *Salix purpurea* ‘Fish Creek’ could perform better than *Salix miyabeana,* ‘SX67’, in situ. Therefore, we suggest implementing an *S. purpurea*—perennial herbaceous co-cropping strategy at this decade-long-abandoned contaminated site or at similar disrupted landscapes.

## 1. Introduction

Among the different types of pollution resulting from human activities, industrial negligence plays a large part in the deterioration of the environment. Not only do industrial facilities affect their immediate surroundings but contamination can extend to much more distant locations [1]. The contamination of otherwise preserved areas occurs through atmospheric or aquatic contamination and because of the improper storage and transportation of hazardous substances [2,3] According to the European Environment Agency [4], about 10% of surface soil contamination results from the spillage of chemicals and trace elements during transport.

In Canada, the Environmental Protection Act [5] requires environmental emergencies to be reported and duly managed. However, immediate control procedures—such as the clean-up that was carried out in the heart of the Bas-Saint-Laurent agricultural region at Saint-Octave-De-Métis (QC, Canada) after a derailed freight train spilled Zinc (Zn) in 2004—cannot exclude the risk of the contamination of the surrounding area. In fact, the complete remediation and long-term monitoring of the nearby arable lands was considered impractical and was, therefore, neglected, as happens all too often [6,7].

Indeed, conventional remediation methods are prohibitively expensive and not fully appropriate in the context of marginal lands adjacent to agricultural sites [8]. Comparatively, phytoremediation is a more viable option, as it takes advantage of the natural properties of plants, and associated microorganisms, to extract or degrade soil contaminants [9]. Besides a high level of societal acceptance, it also has the advantage of being up to ten-fold cheaper than regular physical or chemical techniques [10] and is also particularly well-suited to the management of degraded and moderately contaminated soils such as the large post-incident site at *Saint-Octave-De-Métis* [11]. Obvious environmental impacts associated with intrusive remediation activities are reduced with “green” alternatives and the implementation of a well-planned revegetation program is a rather valuable ecological asset [12]. In addition to reducing environmental hazards through contaminant removal and limiting soil erosion and/or local immobilization, phytoremediation can further improve soil quality and regenerate landscape fertility—two additional environmental services that should be proactively maintained [13,14]. For example, the successful phytomanagement of a large mine-spill site in the Guadiamar Valley (Spain) led to the noteworthy development of the Green Corridor program in the late 1990s, now a 55 km^2^ biodiversity hotspot [15,16,17]. The number of similar projects and small-to-large scale trials keeps rising, thereby allowing phytoresearchers, state regulators, and end-users to design a sustainable future faster and more adequately.

In that respect, feasibility studies under controlled conditions are necessary to minimize the degree of uncertainty regarding long-term outcomes and ensure successful site clean-up [18,19]. the optimal selection of plants [20,21] and management techniques [22,23] are crucial points to be addressed, especially where harsh climatic conditions result in the challenges of soil pollution, as is the case for a train derailment site that is located in a plant zone with a hardiness of 4a (minimum temperature of −34.4 °C to −31.7 °C [24]; growing season length < 140 days [25]).

Conceptually, hyperaccumulating plants are ideal for high-profit commercial applications (phytomining); however, they are rarely productive enough to be suitable for large-scale revegetation schemes [26,27]. Only twenty Zn-hyperaccumulator species are reported in the Global Hyperaccumulator Database [28], none of which grow in our northern latitudes. Therefore, metal-tolerant, low-maintenance, and high-biomass-producing species or species combinations are recommended, especially since their phytoremediation ability can be further enhanced through induced phytoremediation [29,30,31]. However, even the use of so-called environmentally friendly chelating agents such as nitrilotriacetatic acid (NTA) is far from perfect and should be considered very carefully to avoid further leaching of metal–chelate complexes and symptoms of toxicity in plants [23,32,33,34]. Accordingly, as they are adapted to the harsh northern climatic conditions, several plant candidates have been identified in the literature [35,36,37,38,39,40]. In a recent field study [41], *Salix purpurea* ‘Fish Creek’ and *S. miyabeana* ‘SX67’, which were initially selected as energy crops, phytoextracted a significant amount of Zn after three years of growth in polluted soil in Valcartier, Québec, Canada. The properties regarding revegetation ability and contamination tolerance possessed by forage species such as *Festuca arundinacea* [42] and *Medicago sativa* [43] or of other herbaceous species with dense root systems are also well documented, for example, *Andropogon gerardii* [44], *Avena sativa* [45], *Deschampsia cespitosa* [46], *Elymus spp.* [47], *Festuca rubra* [48], *Lolium multiflorum* [49], *Panicum virgatum* [50], *Poa pratensis* [51], and *Spartina pectinata* [52,53]. Purposely designed for the stabilization of disrupted landscapes and retrogressed soils, an affordable commercial seed mixture of the latter herbaceous species could, therefore, be used in the context of a phytoremediation program such as the one that should be deployed in the bare soil at *Saint-Octave-De-Métis*. 

The species-specific and complementary properties of plants growing in contaminated soil with or without the implementation of biodegradable synthetic chelator amendment must be evaluated before the complete design of phytomanagement schemes to limit further detrimental secondary impacts and optimize phytoremediation efficiency. We conducted an experiment under semi-controlled conditions to evaluate the phytoremediation ability of different plant species growing in moderately Zn-contaminated soil excavated fifteen years after a spillage incident and its initial cleanup. The main objectives were to (1) find an efficient phytoremediation strategy to depollute/confine Zn-contaminated soil; to (2) screen *S. purpurea*, *S. miyabeana*, *M. sativa*, *F. arundinacea,* and a commercial grass mix for their growth and Zn uptake effectiveness in that context; and, finally, to (3) investigate the effect of NTA applications on plants and soil. The three-month study measured changes in Zn concentration in the shoots, dry shoot biomass, and willow height and chlorophyll content. Changes in Zn soil content were also carefully monitored.

## 2. Results

### 2.1. Zn Removal in Rhizospheric Soil

Phytoextraction was effective in the soil zone proximal to the roots of all the tested plants. At the end of the experiment, there was no interaction between the two factors *plant species* × *soil treatment* (*p*-value = 0.897) and the effect of the NTA treatment was not significant (*p*-value = 0.267); however, while the total Zn content had only changed by 1.5% (6 mg kg^−1^) in the unplanted pots, it was reduced by >11% (47 mg kg^−1^) in the planted pots (Table 1A, *p*-value < 0.001). Interestingly, there was no difference regarding Zn removal between plant species per se, even if we observed a much greater variability of the total Zn removal in the root zone of willows compared to the other species; both the smallest (8 mg kg^−1^) and the largest (85 mg kg^−1^) changes in Zn concentration were found in pots planted with willow cuttings.

The two factors *plant species* and *soil treatment* interacted to influence the evolution of soil-bioavailable Zn during the experiment (Table 1B, *p*-value < 0.001). While it remained unchanged in the control pots (no plants and no amendment), the bioavailable Zn content shifted when the pots were planted from 1.5 mg kg^−1^ at the beginning of the experiment to as low as 0.5 mg kg^−1^ (>60% removal) in the soil zone proximal to the roots of *F. arundinacea* and the commercial mix without the addition of NTA. The level of bioavailable Zn also reduced in the unplanted pots treated with NTA and in the root zone of *F. arundinacea*, *M. sativa* or the commercial mix, but to a lesser extent (compare data with/without NTA treatment Table 1A); it even increased >2.1–2.3 mg kg^−1^ in the pots planted with willow trees (>50% increase versus initial values).

### 2.2. Plants’ Establishment in Zn-Contaminated Soil

**Figure 1:**
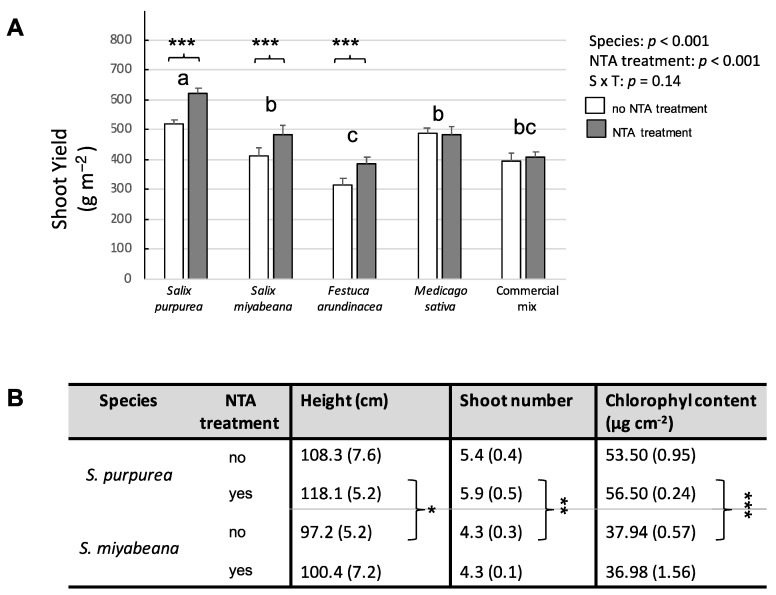
Establishment of tested species on Zn-contaminated soil without/with the addition of NTA as a chelating agent. (**A**) Aboveground yield. White bars represent plants growing in Zn-contaminated soil not treated with NTA; grey bars represent plants growing in Zn-contaminated soil treated with NTA. Values are means ± SE. There was no interaction between the two factors *plant species × soil* treatment; different letters indicate significant differences (Tukey HSD; *n* = 10 between plant species); asterisks indicate a significant effect of NTA treatment (Student’s *t*-test; *n* = 5). (**B**) Growth and physiological parameters of *Salix purpurea* and *Salix miyabeana*. Plant height (cm), shoot number, and chlorophyll content (µg cm^−2^) were recorded at the end of the experiment for the two tested willow species. For all parameters, there was no interaction between the two factors ‘*plant species*’ × ‘*soil treatment*; the ‘*plant species*’ factor, but not ‘*NTA treatment*’, affected height, shoot number, and chlorophyll content. Asterisks indicate significant differences between species (Student’s *t*-test, * *p* < 0.05, ** *p* < 0.01 and *** *p* < 0.001 for height, shoot number, and chlorophyll content, respectively; *n* = 10). Values are means ± SE, ns = non-significant.

A number of parameters were used to evaluate the plants’ establishment in soil excavated from *Saint-Octave-De-Métis* and to assess the effects of the NTA treatment on growth and physiology. First, we carefully monitored the willow cuttings’ survival and the herbaceous species’ germination rate. Mortality occurred only marginally, as seen in Figure 1B. The willows’ establishment and that of the herbaceous species are best described in terms of quantifiable traits such as plant biomass production.

Regarding plant yield, there was no interaction between the two factors *plant species × soil treatment*, but the tested species differed significantly at the end of the experiment (Figure 1A, *p*-value < 0.001). After two months of treatment, *Salix purpurea* ‘Fish Creek’ produced a substantially greater level of shoot biomass than all the other plants, while the commercial mix of herbaceous species and *Festuca arundinacea* was the least productive with 30% and 38% less biomass, respectively, than the willows, with *S. purpurea* producing up to 622 g m*^−^*^2^ of biomass in the soil treated with NTA. Furthermore, the application of the chelating agent was beneficial to the growth of the two willows and to *F. arundinacea,* while it did not affect *Medicago sativa* nor the overall production of the commercial mix (Figure 1A, *p*-value < 0.01); the yield of *S. purpurea*, *S. miyabeana*, and *F. arundinacea* growing in the soil treated with NTA increased by 105, 70, and 70 g m^−^^2^. 

Other growth and physiological traits were thoroughly investigated with respect to the two willow species (Figure 1B). There were significant differences between the two regarding plant height (±15 cm, *p*-value < 0.05), shoot number (±1.4, *p*-value < 0.01), and total chlorophyll content (±18 µg cm*^−^*^2^, *p*-value < 0.001). *S. purpurea* seemed to benefit more from NTA application than *S. miyabeana,* but no statistically significant differences were observed. For example, compared to the non-treated pots, the height of *S. purpurea* planted in the NTA-treated pots increased by 10 cm, whereas it was only 3 cm higher for *S. miyabeana* with NTA amendment compared to the plants growing in the control soil. When the chelating agent was used, shoot diameter and shoot number also slightly increased for *S. purpurea*, but not for *S. miyabeana*. Indeed, the interaction between the two factors *plant species × soil treatment* marginally affected chlorophyll content (*p*-value = 0.06). The leaves of *S. purpurea* growing in the NTA-treated soil had a chlorophyll content 5.6% higher than that of the plants growing in the untreated substrate, while the chlorophyll content of the leaves of *S. miyabeana* was 2.6% lower with the NTA treatment compared to no treatment.

### 2.3. Zn in Aboveground Plant Tissues

**Figure 2:**
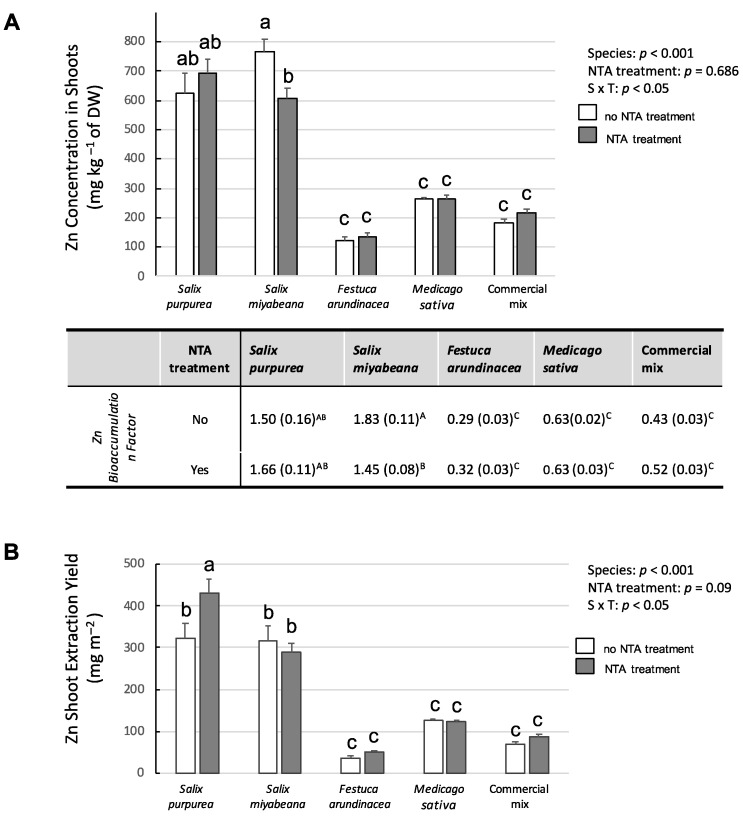
Zn in aboveground tissues of tested species grown in Zn-contaminated soil without/with the addition of NTA as a chelating agent. (**A**) Zn concentration in mg.kg^−1^ and Bioconcentration Factor calculated as the ratio of shoot/soil concentration. (**B**) Zn shoot extraction yield in mg. m^−2^. White bars represent plants growing in Zn-contaminated soil not treated with NTA; grey bars represent plants growing in Zn-contaminated soil treated with NTA. There are interactions between the two factors *plant species × soil treatment*; values are means ± SE. Different letters indicate significant differences (Tukey HSD; *n* = 5).

Regarding the plants’ Zn concentrations, there was an interaction between the two factors *plant species* × *soil treatment* (Figure 2A, *p*-value = 0.05). The concentration of Zn in the willow shoots indicated a Bioconcentration Factor (BCF) > 1.5; this was three times more concentrated than in the shoots of *M. sativa* (BCF = 0.5) or in those of the commercial mix (BCF = 0.6) and up to five times more than in *F. arundinacea’s* aboveground tissues (compare 765 mg kg^−1^ to 122 mg kg^−1^ of dry matter), for which the Zn was concentration was the lowest (BCF = 0.3). The NTA amendment negatively affected the Zn concentration in *S. miyabeana’s* aboveground biomass; no significant changes were observed for the other species.

With respect to the Zn extraction yield, there was also an interaction between the two factors *plant species* × *soil treatment* (Figure 2B). The extraction profiles for the herbaceous species were comparable to what was observed for the Zn shoot concentration; however, the highest extraction yield (four times higher) was not observed in *S. miyabeana* but for the *S. purpurea* trees growing in the soil treated with NTA (>400 mg m^−2^; 300 mg m^−2^ in all other willow trees). No other species were affected by the application of NTA. 

## 3. Discussion

The present study helped us quickly determine a phytoremediation strategy that should be appropriate at *Saint-Octave-De-Métis* and in contaminated sites with similar characteristics. In less than 100 days, the herbaceous species and willow trees grown under semi-controlled conditions demonstrated high Zn phytoremediation potential.

### 3.1. Soil Remediation

Even if the measurement of a significant decrease in bulk soil metal content was neither in the scope of the present study nor expected after a single growing season [54,55,56], the careful monitoring of the Zn content in a rhizospheric substrate was much informative with respect to understanding the different mechanisms in place during this preliminary pot trial. In fact, the need for a second phase of remediation was confirmed by the initial measurements of the Zn concentration that far exceeded the legal threshold. Moreover, the slight depletion in the total metal concentration we measured at the end of the experiment in the unplanted pots verifies our concerns regarding potential runoff towards adjacent fields. According to Garcia et al. [57], a large amount of Zn in soil can be gradually released into a soil solution. 

On the other hand, the results were encouraging since land left in a fallow condition for over a decade could readily benefit from cultivation. The decrease in Zn content was significantly stronger in the soil directly in contact with root systems than in the control pots, thus showing—with respect to plant growth and the context—the efficiency of the tested crops in terms of Zn phytostabilization and metal uptake. No tangible differences regarding total Zn removal were measured between the rhizospheres of the species tested. However, as expected, the addition of the chelant was able to increase Zn solubility and further modify the Zn equilibrium of this long-term-contaminated substrate, impairing the apparent removal of bioavailable contaminants for all except the willow species, for which this fraction even greatly increased in the rhizosphere, i.e., the roots appeared to be unable to locally compensate the substantial expansion of the Zn mobile fraction. These results revealed very distinct but complementary patterns of influence on the local soil properties. Indeed, willows are well known to extract large quantities of Zn [58] while herbaceous species have a high tolerance to contamination, although possessing lower phytoextraction capacity [43,59,60]. Therefore, we suggest that *F. arundinacea*, *M. sativa,* and the commercial mix can stabilize the immediate roots in the surrounding area and that the root system of the two water-demanding willows could have a stronger spatial influence and the ability to drain soil solutions from a larger volume. In addition, even though the mobile form of Zn rose in the tree rhizospheres, the environmental risk due to NTA application should be rather limited considering the minimal dosage we used [61,62] and the transient nature of the metal–NTA complexes [63], especially if willows and herbaceous species are cultivated together to maximize remediation potential [64].

### 3.2. Evaluation of Plant Species Candidates

All the tested species showed no significant level of mortality and good adaptation to the soil excavated from *Saint-Octave-De-Métis* despite the poor substrate quality and excessive Zn content. In accordance with a previous study on contaminated soil [65], we observed high yield differences between crops having an otherwise similar growth potential under optimal conditions (Laurent et al., 2015). Not surprisingly, the elite cultivar *Salix purpurea* ‘Fish Creek’ ranked first in this context and should be considered of particular value for the restoration of field sites. Indeed, it was selected for its high-performance across diverse field conditions [66] and because Courchesne et al. [41] had also determined its superiority over the high-yielding biomass of *S. miyabeana* ‘SX67’ during a three-year long phytoremediation field trial in southern Québec. 

Among herbaceous species, the ‘nitrogen-fixer’ *Medicago sativa* produced significantly more shoots than *Festuca arundinaceae,* which was the poorest-performing species we have tested, and the same is true for the commercial mixture. Even if vegetation characterization and nodule number were not determined, as it was off-topic, we can assume that rhizobium–legume symbiosis and/or species diversity could have de facto resulted in the alleviation of stresses caused by the relative lack of resources [67] or by Zn contamination [64,68]. Furthermore, since amendment with a chelating agent is known to increase both the availability of metal ions as well as nutrient mobility, it is noteworthy that the NTA application did not have any influence on the aboveground growth of *M. sativa* or of the commercial mix, hence highlighting the overall resilience of the two tested crops [69]. 

On the contrary, the willow trees and *F. arundinacea* benefitted from the NTA application. As stated above, similar results have already been reported [70,71] for different plant species, among which were *F. arundinacea* [31] and *Salix* cultivars. In our case, *S. purpurea* ‘Fish Creek’ was the most responsive to the NTA application, while Zhivotovsky et al. [72] found a chelator-related growth benefit for the *S. purpurea* cultivar ‘Allegany’ growing in Pb-contaminated soil but not for *S. miyabeana* ‘SX64’, ‘SX61’, nor ‘SX67’. Along the same lines, the other recorded growth and physiological parameters indicated that *S. purpurea* was a better fit during this preliminary trial: the plant height, shoot number, and chlorophyll content were higher than in *S. miyabeana*, and they also tended to increase with the addition of NTA whilst the opposite occurred for *S. miyabeana’s* chlorophyll content. As chlorosis and retarded growth are phytotoxic symptoms caused by Zn [73], one could suspect that *S. miyabeana* had a lower tolerance to the soil excavated from *Saint-Octave-De-Métis*.

## 4. Conclusions

### What Fits Best

Although cumbersome at first, the analysis of plant Zn content was undoubtedly valuable and has helped us in various ways to finally narrow down an optimal phytoremediation strategy. Although the degrees of contaminant removal in the rhizospheres were similar, the Zn content in the aboveground biomass differed strongly between species. This result per se confirmed the respective potentials of willows and herbaceous species for phytoextraction and phytostabilization in situ [74].

Furthermore, this experimental design under semi-controlled conditions allowed us to widen the scope of the present study. From one single homogenized substrate, we were able to investigate the plant response to two levels of bioavailable Zn contamination (i.e., with/without NTA)—an interesting proxy of the dynamic and heterogenous conditions encountered by crop roots in the field [75]. In fact, almost no plant benefited from treatment with the chelating agent in terms of Zn concentration—it even resulted in an impaired uptake for *S. miyabeana*. Therefore, we were able to reasonably speculate that the plant shoots had already reached the maximum viable Zn content [76,77,78], all the more so because Desjardins et al. in their work under comparable conditions [64] reported very similar Zn concentrations for *S. miyabeana*, *F. arundinaceae,* and *M. sativa* using a substrate doubly less contaminated. 

Finally, in light of the physiological data and the monitoring of the Zn content in the plant tissues, the slight increase in the *S. purpurea* extraction yield in the presence of the chelant may be more related to the nutritional bioavailability improvement of the soil than to the contaminant per se. Concerning the use of NTA, which was impractical and irrelevant considering the lack of positive outcome revealed by this preliminary experiment, our suggestion would be to implement an *S. purpurea*-perennial herbaceous co-cropping strategy at the decade-long abandoned brownfield at *Saint-Octave-De-Métis* [68,79]. Although it requires relatively frequent coppicing, yielding a valuable biomass product nevertheless, to avoid contaminant entry in the food chain [80,81,82], this phytomanagement scenario that combines phytoextraction, phytostabilization, and phytorestoration is non-labor-intensive and would benefit local agrobiodiversity [37,83,84,85] (Futughe et al., 2020; Jacklin et al., 2021; Kuzovkina and Quigley, 2005; Mosseler et al., 2014).

## 5. Materials and Methods

### 5.1. Experimental Design

**Figure 3:**
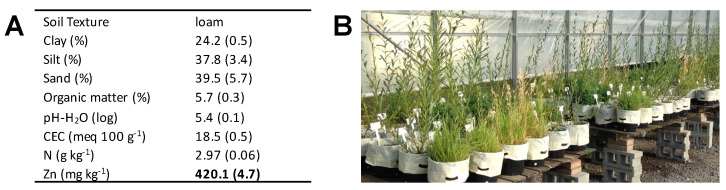
Phytoremediation experiment concerning Zn-contaminated soil excavated from an area polluted by a freight train derailment and spill incident (**A**) Soil’s initial physical and chemical characteristics. Values are the mean ± SE (*n* = 5). Numbers in bold exceed Canadian criteria of trace element soil concentration for residential land use. CEC = cation exchange capacity; (**B**) Partial view of the open greenhouse experiment after 90 days of growth.

In May 2017, an experiment was set up in the open-air greenhouse facility of the Montréal Botanical Garden (Montréal, QC, Canada). Thirty-centimetre-diameter plastic pots sealed with a plastic membrane to avoid water runoff were used and filled with eight litres of contaminated soil. The properties of the homogenized soil are depicted in Figure 3A and indicate Zn concentration exceeding Canadian criteria for both agricultural and residential land use for this substrate collected from the site of a freight train derailment and spill in *Saint-Octave-De-Métis*.

To assess the phytoremediation potential of different plant species and the effects of a chelating agent on their phytoextraction efficiency, two willow species (*Salix purpurea* ‘Fish Creek’ and *S. miyabeana* ‘SX67’), two grasses (*Medicago sativa* and *Festuca arundinacea*), and one commercial herbaceous mix (comprising *Andropogon gerardii*, *Avena sativa*, *Deschampsia cespitosa*, *Elymus canadensis*, *Elymus trachycaulus, Festuca rubra*, *Lolium multiflorum*, *Panicum virgatum, Poa pratensis*, and *Spartina pectinata*) were tested and/or treated with nitrilotriacetic acid (NTA).

The randomized block experimental design consisted of 60 experimental units (5 plant species + 1 control (non-planted)) × (2 NTA treatments (with/without)) × 5 replicates or blocks). One willow cutting was planted, or the equivalent of 15 kg ha^−1^ of grass seeds were sown per pot (Figure 3B). Plants were regularly watered throughout the experiment with tap water in order to maintain a good moisture content. NTA treatment began 30 days after plantation: to maintain an effective and stable soil concentration, NTA was applied every 5 days at a dose of 2 mmol per kg of soil; the pots that did not receive the chelating agent were given an equivalent volume (50 mL) of tap water.

### 5.2. Measurement Strategy, Soil and Plant Sampling, and Analyses

To evaluate the Zn phytoextraction ability and the effectiveness of the application of a chelating agent in the tested conditions, aboveground plant biomass, height, shoot number, leaf chlorophyll content (portable chlorophyll meter, atLeaf+), and plant-tissue Zn concentration were measured. Zn removal in soil (Zn removal = initial Zn concentration in soil—final Zn concentration in soil), Zn extraction yield (EY = above-ground plant biomass x concentration in aboveground plant biomass), and aboveground bioconcentration factor (BCF = metal concentration in plant/initial metal concentration in soil) were also determined. Soil samples were collected at the beginning of the experiment (t_i_); at 90 days after plantation, the soil was sampled again (t_f_), and the plants were harvested. Fresh aerial parts were oven-dried at 70 °C for 48 h to determine biomass dry weight (DW) and calculate yield.

The total (acid-recoverable) concentrations were determined in soil and plant tissues. Bioavailable (water-soluble) fraction of Zn was also analyzed. Zn content in soil (t_i_, t_f_) was determine by Inductively Coupled Plasma-Mass Spectrometry (NexION 300× ICP-MS Spectrometer, Perkin Elmer, Waltham, MA, USA). For the bioavailable fraction, 4 g of dried and sieved (<500 µm) samples was 10^1^ diluted in ultrapure water and placed into 50 mL tubes; then, the 50 mL tubes were shaken for 2 h, centrifuged at 1400× *g* for 15 min, and the supernatant was filtered through 0.45 µm nylon membrane to remove residual particles before a 15 mL mineralized filtrate acidified with 0.04 mL of HNO_3_ was added (50%, *v/v*). For the total Zn concentration in soil and plant tissues, 200 mg of ground dry material was digested with hot HNO_3_ according to Wilson et al. [86].

### 5.3. Data Analyses

All the statistical analyses were carried out using R software (R Development Core Team., Vienna, Austria, 2008) and SAS JMP v.9.0 (SAS Institute Inc., Cary, NC, USA, 2012). Missing data (t0 soil samples (3)) were generated with MICE R package (Multivariate Imputation by Chained Equations, available online: http://multiple-imputation.com, accessed on 13 December 2022). Complete transformations were performed when needed to respect normality and homoscedasticity assumptions; log transformations were performed with dry biomass and extraction yield data. Data were subjected to a two-way analysis of variance (plant species × NTA treatment) followed by post hoc test when significant (between plant species: Tukey’s HSD; between NTA treatments: Student’s *t*-test). Finally, a Dunnett’s post hoc test was performed on the differential analysis of t_0_ and t_1_ soil Zn concentration variables. *p* ≤ 0.05 was used as significance level in all analyses.

## Figures and Tables

**Table 1:** Total and bioavailable Zn removal at the end of the experiment. Values are means ± SE (in parenthesis). Different letters indicate significant difference (Tukey HSD, *p* < 0.001, *n* = 5). (**A**) Regarding Total Zn removal, there was no interaction between the two factors of plant species × soil treatment; (**B**) there was an interaction between the two factors—plant species × soil treatment—with respect to bioavailable Zn removal.

		NTA Treatment	Non Planted		*Salix purpurea*		*Salix miyabeana*		*Festuca arundinacea*		*Medicago sativa*		Commercial Mix	
**A**	Total Zn removal (mg kg^−1^ of soil)	No	0.98 (3.02)	B	33.24 (11.79)	A	27.40 (9.17)	A	48.62 (3.64)	A	53.98 (5.95)	A	36.55 (7.56)	A
		Yes	10.38 (5.57)		37.19 (4.64)		46.55 (10.19)		56.42 (4.47)		56.03 (5.44)		42.75 (7.21)	
**B**	Biodisponible Zn removal (mg kg^−1^ of soi)	No	0.08 (0.32)	BC	0.60 (0.14)	AB	0.48 (0.05)	AB	0.96 (0.08)	A	0.60 (0.08)	AB	0.95 (0.05)	A
		Yes	0.64 (0.19)	AB	−0.58 (0.21)	CD	−0.81 (0.24)	D	0.55 (0.13)	AB	0.10 (0.21)	BC	0.47 (0.08)	AB
